# Relationship between obsessive personality traits and eating disorders

**DOI:** 10.1007/s40519-026-01829-5

**Published:** 2026-03-12

**Authors:** María J. Triguero-López, M. Inmaculada Moreno-García, Manuel Morales-Ortiz

**Affiliations:** 1 Department of Personality, Psychological Assessment, and Treatment, Faculty of Psychology, University of Seville, Calle Camilo José Cela, 41018 Seville, Spain; 2 Department of Experimental Psychology, Faculty of Psychology, University of Seville, Calle Camilo José Cela, 41018 Seville, Spain

**Keywords:** Eating disorders, Obsessive personality, Perfectionism, Cognitive flexibility, Desire for control

## Abstract

This study aimed to examine associations between obsessive personality traits (perfectionism, desire for control, and cognitive flexibility) and eating-related symptomatology in a non-clinical sample of young adults. The objective was to explore which subdimensions were most strongly associated with eating attitudes. A cross-sectional study was conducted with 890 students aged 18–25 years, who completed validated self-report measures: the Eating Attitudes Test (EAT-26) (Garner and Garfinkel in Psychol Med 9(2):273–279, 1979), the Frost Multidimensional Perfectionism Scale (FMPS) (Frost et al. in Cognit Ther Res 14(5):449–468, 1990), the Desirability of Control Scale (Burger and Cooper in Motiv Emot 3(4):381–393, 1979), and the Personalised Psychological Flexibility Index (PPFI) (Kashdan et al. in Psychol Assess 32(9):829–850, 2020). Regression analyses were conducted controlling for sex and age. The results indicated statistically significant but modest associations between several subdimensions of perfectionism, cognitive flexibility, desire for control, and eating-related symptomatology. These findings expand the previous evidence on obsessive personality traits in relation to eating behaviours and should be interpreted as exploratory and correlational in nature.

*Level of evidence*: 2 (observational, cross-sectional study).

**Supplementary Information:**

The online version contains supplementary material available at 10.1007/s40519-026-01829-5.

## Introduction

Eating disorders (EDs) are characterised by persistent alterations in behaviours related to eating (American Psychiatric Association [144]). They represent a global public health issue, with higher prevalence among women and young populations [28, 59, 66, 80, 81, 125, 128, 142]. EDs tend to follow a chronic course, with high comorbidity and elevated mortality rates [35, 55].

Despite advances in identifying risk factors, the complexity of the phenomenon calls for more integrative approaches [85, 114]. Among the psychological factors involved, obsessive personality traits such as perfectionism, cognitive rigidity, and desire for control have demonstrated reliable associations with both the risk and severity of EDs [43, 51, 117]. These traits are core components of obsessive–compulsive personality pathology and are linked to mechanisms, cognitive inflexibility, heightened perfectionism, and maladaptive control strategies, that sustain disordered eating behaviours [36, 70, 91, 113]. Nevertheless, theoretical models integrating these factors remain limited [26, 62, 85]. Recent frameworks suggest that examining obsessive personality traits jointly provides a clearer account of their combined contribution to ED psychopathology, in line with cognitive-interpersonal models of anorexia nervosa and longitudinal evidence linking early obsessive traits to adult ED symptoms [45, 135]. Despite their substantial conceptual and empirical overlap, these traits have often been studied independently, constraining understanding of their concurrent and relative associations with eating-related symptoms [77]. Emerging evidence supports a profile-based approach, suggesting that specific constellations of obsessive and inflexible traits are more informative than single traits in isolation [16, 113].

The relationship between the personality dimension of perfectionism and EDs has also been widely studied [7, 10, 30, 78, 123]. Given that perfectionism is a multidimensional construct, it has been observed that each dimension may be either positively or negatively related to mental health outcomes [61, 73].

Cognitive flexibility (CF), understood as the capacity to adapt effectively to changing demands, is a key multidimensional construct in understanding the psychological processes involved in eating disorders. At its extreme, low cognitive flexibility can be conceptualised as a trait of inflexibility, entailing greater rigidity in information processing and behavioural response [21, 48, 62, 74, 109]. Its relevance has been highlighted in therapeutic models such as Acceptance and Commitment Therapy [53, 130], where it is associated with enhanced emotional regulation and reduced symptomatology. Recent studies have shown its relation to internalisation of the thin ideal and symptom maintenance in anorexia nervosa, particularly across age [9, 22, 27, 86, 95]. Furthermore, therapeutic intervention targeting CF may positively influence prognosis and treatment of EDs [54, 56, 92, 104].

Beyond CF, another relevant trait in the psychopathology of eating disorders is the desire for control (DC). Initially conceptualised as unidimensional by Burger and Cooper [15], it has evolved into a multidimensional structure comprising: general desire for control, avoidance of dependency, and control over preparation and task prevention [17, 39, 40, 44, 111, 133, 147]. General desire for control refers to the need to influence one’s environment [15, 40]; avoidance of dependency refers to a preference for self-sufficiency, particularly pronounced in patients with anorexia nervosa [33, 115]; and control over preparation implies meticulous planning and a drive for predictability [76, 120].

This conceptual complexity challenges research, as different control dimensions may operate differently depending on ED type or individual psychopathology [120]. Emotional aspects, such as fear of losing control and feelings of ineffectiveness, are significant predictors of disordered eating and obsessive–compulsive symptoms [34, 101]. Intolerance of uncertainty, linked to DC, is a transdiagnostic vulnerability factor in EDs and other disorders [6].

While previous studies examined perfectionism, CF, and DC separately, this study evaluates these traits simultaneously in a non-clinical sample. This allows examination of their relative and combined associations with eating-related behaviours, providing a more integrative perspective on obsessive personality profiles and their relevance to early disordered eating, complementing prior research focused on single traits or clinical populations.

In light of the above, the aim of this study was to analyse the relationship between obsessive personality traits-perfectionism, DC, and CF and ED-related behaviours in a non-clinical sample. Three specific objectives were established. (1) To examine the relationship between the level of perfectionism and ED-related behaviours, based on its six subdimensions [41]: concern over mistakes, personal standards, parental expectations, parental criticism, doubts about one’s own actions, and organisation. (2) To analyse whether CF levels are associated with ED-related behaviours, considering its three dimensions [62]: avoidance, control, and acceptance. To explore whether DC level is linked to ED-related behaviours, focusing on three dimensions [15]: general desire for control, avoidance of dependency, and control over preparation/prevention of tasks.

Based on the literature, hypotheses (H) were proposed and grouped into three dimensions: perfectionism, CF, and DC. Regarding perfectionism, it is expected that concern over mistakes (H1.1), high personal standards (H1.2), elevated parental expectations (H1.3), perceived parental criticism (H1.4), a tendency to doubt one’s own actions (H1.5), and a high level of organisation (H1.6) will be positively associated with ED-related behaviours. In CF, it is hypothesised that higher levels of avoidance (H2.1) and need for control (H2.2) will be associated with greater ED-related behaviours, while acceptance (H2.3) will be negatively associated. Finally, in DC, it is proposed that general desire for control (H3.1), avoidance of dependency (H3.2), and control over preparation/prevention of tasks (H3.3) will be positively associated with ED-related behaviours (see Table 1).

**Table 1:** Research hypotheses

Hypothesis	
1.1	Concern about mistakes will be positively associated with ED-related behaviours
1.2	High personal standards will be positively associated with ED-related behaviours
1.3	High parental expectations will be positively associated with ED-related behaviours
1.4	Perceived parental criticism will be positively associated with ED-related behaviours
1.5	Doubts about one’s own actions will be positively associated with ED-related behaviours
1.6	Organization will be positively associated with ED-related behaviours
2.1	Avoidance will be positively associated with ED-related behaviours
2.2	Certainty will be positively associated with ED-related behaviours
2.3	Acceptance will be negatively associated with ED-related behaviours
3.1	General desire for control will be positively associated with ED-related behaviours
3.2	Avoidance of dependence will be positively associated with ED-related behaviours
3.3	Control over task or action preparation/prevention will be positively associated with ED-related behaviours

**Fig. 1:**
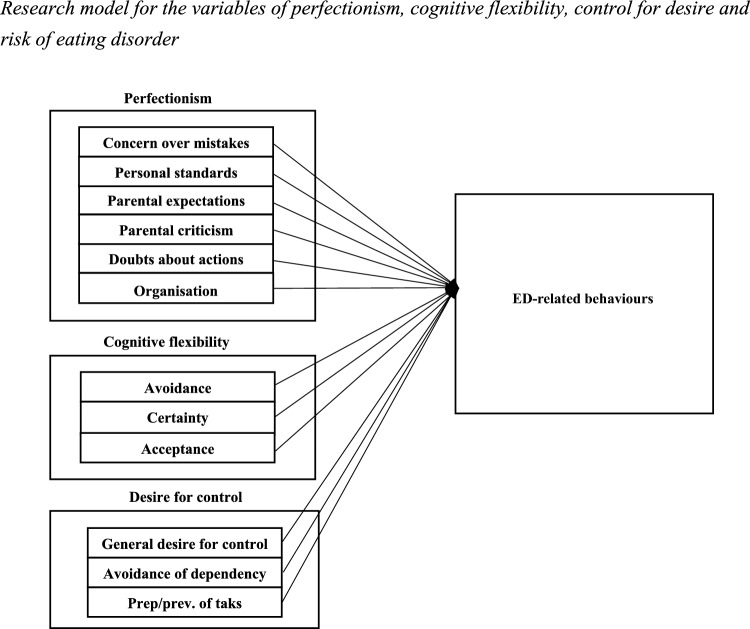
Research model for the variables of perfectionism, cognitive flexibility, control for desire and risk of eating disorder

These objectives guided our research model, as can be observed in Fig. 1.

## Method

### Participants and procedure

Ethical approval was obtained from the Ethics Committee of the University of Seville for research involving human participants. The procedures used in this study adhered to the principles of the Declaration of Helsinki [148].

The current research was a cross-sectional quantitative study conducted in Seville, Spain. The target population comprised young individuals aged 18–25. The sample consisted of 890 students (*M* = 20.02, SD = 1.94) enrolled in intermediate or advanced vocational training or university studies. Of these, 338 identified as female (38%, *M* = 20.21, SD = 1.89) and 546 as male (61.3%, *M* = 19.91, SD = 1.97) (see Table S1 in the supplementary material). The focus was on late adolescents and young adults (Allen et al. [2]), whose personality traits could be considered stable [11], [19], [87]. However, the sample came from a single geographical region and overrepresented males, which may limit generalizability and introduce selection bias, potentially restricting the applicability of the findings to more diverse populations [23, 129].

Inclusion criteria were: age 18–25, enrollment in middle-, higher-, or university-level education, and completion of the assessment protocol. Incomplete or inconsistent questionnaires were excluded.

Each participant was provided with the assessment materials, which included an answer sheet, an information sheet, informed consent form, and all questionnaires. The research procedure was then thoroughly explained. Once all doubts were resolved and both the information sheet and consent form were signed, the participants proceeded to complete the instruments. Data collection lasted approximately 40 min per group. No clinical assessments (e.g., BMI or formal ED diagnoses) were conducted, and all measures were self-reported, limiting conclusions to eating disorder-related behaviours in a non-clinical population.

### Instruments

Eating Attitudes Test (EAT-26) [42] assesses eating-related attitudes and behaviours. It has shown good construct validity for identifying individuals with elevated eating-related concerns. In this study, internal consistency was excellent (*α* = 0.90) and test–retest reliability was *r* = 0.93. The instrument includes 6 Likert response options.

Frost Multidimensional Perfectionism Scale (FMPS) [41] has a strong empirical tradition and significant influence in the study of perfectionism. It has also been validated in Spanish samples, supporting its suitability for this population [18, 38]. Measures six dimensions of perfectionism. Prior research supports its construct and convergent validity, particularly with measures of obsessive traits. Internal consistency ranged from *α* = 0.87 to 0.93.

Desirability of Control Scale [15] has been validated for the Spanish population by Calvo et al. [17], demonstrating adequate psychometric properties for assessing desire for control and its subdimensions [58, 98, 139]. It comprises 20 items with 7 Likert response options. Prior studies indicate adequate construct validity, internal consistency (*α* = 0.736), and test–retest reliability (*r* = 0.713).

Personalised Psychological Flexibility Index (PPFI) [62] is a self-report questionnaire used to assess psychological flexibility in adults. Its overall reliability reached a Cronbach’s alpha of 0.84. The instrument also demonstrates construct validity, with scores correlating as expected with related measures of psychological flexibility [21] (Xia et al. [141]) and includes 7 Likert response options.

Items related to demographic information were also included, specifically sex and age.

### Statistical analysis

Descriptive analyses were carried out using Jamovi software (v. 2.6) [146]. Confirmatory factor analyses (CFA) were conducted to evaluate the measurement model, and linear regression models were estimated using R software (v. 2.4) [145]. The bootstrap technique was employed to validate the regression models [20, 29], as applied in previous studies [94]. This procedure allowed estimating confidence intervals for the distribution of the statistic of interest (BCa interval) without making parametric assumptions. For the calculation of these intervals, missing values were removed.

## Results

Firstly, descriptive data were obtained, including missing values, means, medians, standard deviations, and minimum and maximum scores (Table S2). Correlations and regression models were calculated from total scores for each dimension. The dependent variable was ED-related behaviours, with independent variables including six perfectionism dimensions (concern over mistakes, personal standards, parental expectations, parental criticism, doubts about actions, organisation), three CF dimensions (avoidance, certainty, acceptance), and three DC dimensions (general desire for control, dependency avoidance, control over preparation/prevention of tasks). Sex and age were included as covariates in all models.

Regarding eating symptomatology assessed through the EAT-26, scores indicated a moderate severity level with wide variability across participants (*M* = 31.84, SD = 14.16).

A confirmatory factor analysis (CFA) was conducted for the Desire for Control scale (DESC), using the DWLS estimator (see Table 2). The resulting model consisted of three factors: general desire for control (items 3, 4, and 7), avoidance of dependency (items 1 and 10), and control over preparation/prevention of tasks (items 2 and 9). During the process, item 5 was removed from the analysis due to its low significance and non-exclusivity in any of the factors. A minimum threshold of 0.40 was established for factor loadings; items falling below this threshold were excluded to ensure the validity of the analysis [12]. The model showed a good fit: *χ*^2^(11) = 18.06; *p* = 0.001; CFI = 0.99; TLI = 0.99; RMSEA = 0.030; SRMR = 0.029.

**Table 2:** Parameters of the CFA model for the variable of desire for control

lhs	rhs	est	se	z	*p* value
GDC	DESCON3	0.835	0.025	33.977	0
GDC	DESCON4	0.860	0.026	32.581	0
GDC	DESCON7	0.468	0.034	13.849	0
AD	DESCON1	0.694	0.041	16.873	0
AD	DESCON10	0.809	0.045	18.006	0
CPP	DESCON2	0.576	0.047	12.228	0
CPP	DESCON9	0.621	0.047	13.172	0

Subdimension of the variable of desire for control (DC). General Desire for Control (GDC). Item 3 of the DC questionnaire: DESCON3, Item 4: DESCON4, Item 7: DESCON7. Subdimension of the DC variable. Avoidance of dependency (AD). Item 1: DESCON1, Item 10: DESCON10. Subdimension of the DC variable. Control over Preparation or Prevention of Tasks or Actions (CPP). Item 2: DESCON2, Item 9: DESCON9. Lhs: Left-Hand Side, rhs: Right-Hand Side

**Fig. 2:**
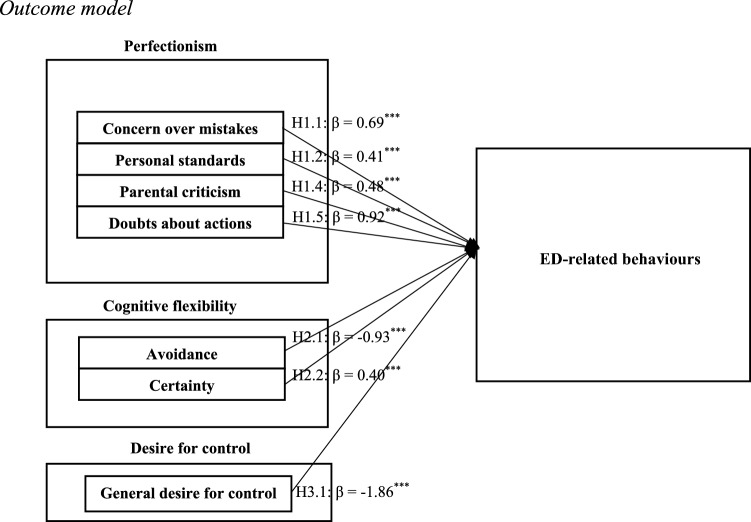
Outcome model. Figure shows the results obtained with the completed regression model, which was statistically significant. The model includes the following independent variables: Perfectionism, Cognitive Flexibility and Control for Desire. The negative beta coefficients for the variables indicate that they have a negative impact on the dependent variable, whereas the positive beta coefficients indicate a positive impact on the dependent variable

Linear regressions were conducted for the subdimensions of perfectionism, CF, and DC against ED-related behaviours (Table 3). All dimensions except organisation, parental expectations, acceptance, dependency avoidance, and control over preparation/prevention of tasks showed significant associations. The full model including only significant variables is shown in Fig. 2.

**Table 3:** Estimators of the regression models and standardised regression coefficients for all the assessed dimensions, with sex and age as control variables

H	Relation	*R*_corrected_^2^	SE	Bca	*F*	*P*	*β* dimension	Sex	Age	Hypothesis accepted
H1.1	CM-ED-related	0.129	0.07	[0.52, 0.86]	35.7	<0.001***	0.69***	−3.54***	0.58*	Yes
H1.2	PS-ED-related	0.087	0.06	[0.27, 0.55]	23.5	<0.001***	0.41***	−4.69***	0.69**	Yes
H1.3	PEX-ED-related	0	0.15	[−0.07, 0.53]	9.24	0.108	0.24	−3.77***	0.73**	No
H1.4	PC-ED-related	0.0749	0.08	[0.30, 0.66]	20.0	<0.001***	0.48***	−3.33***	0.60**	Yes
H1.5	DA-ED-related	0.044	0.28	[0.35, 1.55]	11.9	<0.01**	0.92**	−3.80***	0.71**	Yes
H1.6	ORG-ED-related	0	0.09	[−0.15, 0.22]	8.41	0.687	0.03	−3.67***	0.76**	No
H2.1	A-ED-related	0.031	0.16	[−1.31, −0.58]	19.9	<0.001***	−0.93***	3.03**	0.54*	Yes
H2.2	CER-ED-related	0.054	0.09	[0.21, 0.58]	14.4	<0.001***	0.40***	−4.15***	0.76**	Yes
H2.3	AC-ED-related	0	0.18	[−0.38, 0.36]	8.35	0.965	−0.007	3.72***	0.76**	No
H3.1	GDC-ED-related	0.044	0.48	[−2.91, −0.82]	12.0	<0.001***	−1.86***	3.62***	0.74**	Yes
H3.2	AD-ED-related	0	0.48	[−0.67, 1.37]	8.86	0.226	0.33	3.68***	0.75**	No
H3.3	CPP-ED-related	0	0.49	[−0.95, 0.96]	8.65	0.354	−0.005	−3.72***	0.76**	No

*CM* concern over mistakes, *PS* personal standards, *PEX* parental expectations, *PC* parental criticism, *DA* doubts about one’s actions, *ORG* organisation, *A* avoidance, *CER* certainty, *AC* acceptance, *GDC* general desire for control, *AD* avoidance of dependency, *CPP* control over preparation/prevention of tasks, *Β* standardised regression coefficient

Standard error (SE), hypothesis (H). * *p* < 0.05; ** *p* < 0.01; *** *p* < 0.001

H1.1. Concern over mistakes (*M* = 11.3, SD = 5.97) was positively associated with ED-related behaviours (*β* = 0.69***, *p* < 0.001), explaining approximately 13% of the variance. This pattern supports previous research indicating this relationship [24, 99, 105, 138], where higher concern over mistakes tended to co-occur with higher ED-related behaviours. Sex had a significant effect (*β* = −3.54, *p* < 0.001), with women scoring higher, consistent with prior evidence [121] and classic findings linking maladaptive perfectionism to eating pathology in female samples [4, 100]. Age showed a small but significant effect (*β* = 0.58*), aligning with age-related continuity in internalising symptoms [140]. The correlation between the two variables was 0.311 (see Table S3).

H1.2. Personal standards (*M* = 24.1, SD = 7.59) were positively associated with ED-related behaviours (*β* = 0.41***, *p* < 0.001), echoing prior findings [1, 84, 116]. The effect, while statistically significant, was modest size. Sex (*β* = −4.69***) and age (*β* = 0.69**) were significant covariates. Higher ED-related behaviours among females align with established sex differences in ED prevalence and severity [3, 106], while the small age effect may reflect differences in ED-related concerns across age groups in this sample [93, 140].

H1.3. Parental expectations (*M* = 6.82, SD = 3.25) were not significantly associated with ED-related behaviours (*β* = 0.24, *p* = 0.108), despite previous evidence highlighting the role of family dynamics in ED development [88, 122, 131]. Supporting previous evidence [85, 121], sex (*β* = −3.77***) and age (*β* = 0.73**) remained significant with higher symptom levels in women and modest persistence with increasing age.

H1.4. Perceived parental criticism (*M* = 10.8, SD = 5.75) (for example, the item “As a child I was punished for not doing things perfectly”) was positively associated with ED-related behaviours (*β* = 0.48***, *p* < 0.001) corroborating previous findings [127, 137], where higher parental criticism tended to co-occur with higher ED-related behaviours [68, 108]. This relationship accounted for approximately 7.5% of the variance, indicating a modest effect. Sex (*β* = −3.33***) and age (*β* = 0.60**) remained significant, reflecting prior evidence, with higher scores in women [3] and modest increases with age [140].

H1.5. Doubts about one’s actions (*M* = 4.24, SD = 1.69) (e.g., “In general, I have doubts about what I do”) were positively associated with ED-related behaviours (*β* = 0.92, *p* < 0.01**, adjusted *R*^2aj^ = 0.04), suggesting that higher indecisiveness co-occurred with higher ED-related behaviours [14, 57]. Although statistically significant, this association accounted for a small proportion of the variance. Sex (*β* = −3.80***) and age (*β* = 0.71**) remained significant, with women showing higher scores [69], and a small age-related persistence, suggesting modest but consistent demographic effects [85].

H1.6. No significant association was found between organisation level (*M* = 20.9, SD = 5.28) and ED-related behaviours, and thus the null hypothesis could not be rejected. While some previous studies have reported significant associations [123, 137], others suggest that these associations are weak or non-existent [103]. Sex (*β* = −3.67***) and age (*β* = 0.76**) remained significant with higher scores in women and a small age-related increase, supporting established demographic patterns in ED symptomatology [85].

H2.1. Avoidance (e.g., “When I feel stressed pursuing a goal, I give up”) (*M* = 10.2, SD = 3.03) was negatively associated with ED-related behaviours (*β* = −0.93***, *p* < 0.001), in line with previous studies linking avoidance and cognitive rigidity [49, 50, 102, 107]. Although statistically significant, the association was small. Sex (*β* = 3.03**) and age (*β* = 0.54*) remained significant, with higher ED-related behaviours in women [121], and a modest age effect, in accordance with evidence that ED onset peaks in young adulthood but may persist or recur into later life [140].

H2.2. Certainty was positively associated with ED-related behaviours (*β* = 0.40***, *p* < 0.001), consistent with prior literature [27, 132, 134]. Although statistically significant, the effect was small. Sex (*β* = −4.15***) and age (*β* = 0.76**) were significant, with female participants and older participants reporting higher ED-related behaviours, in accordance with evidence of the persistence of ED traits across adulthood [5, 85].

H2.3. Acceptance (*M* = 9.60, SD = 2.67) did not significantly predict ED-related behaviours (*β* = −0.007, *p* = 0.965). This result aligns with recent research suggesting the multidimensional complexity of this construct and the need to examine its components separately. For instance, Cherry et al., [21] stated that it is a multidimensional construct, with different dimensions potentially affecting mental health differently. Moreover, Berner et al. [9] and Clarke and Kiropoulos [22] found that age and sex may moderate the effects of CF, which could explain the significant associations found in these sociodemographic variables in our study. Sex (*β* = 3.72***) and age (*β* = 0.76**) were significant, suggesting that demographic factors may influence ED-related behaviours independently of acceptance levels [23, 140].

H3.1. General desire for control (*M* = 5.80, SD = 1.04) was negatively associated with ED-related behaviours (*β* = −1.86***, *p* < 0.001), with sex (*β* = 3.62***, *p* < 0.001) and age (*β* = 0.74**, *p* = 0.003) also significant. Higher scores in females and modest increases with age tended to co-occur with ED-related behaviours [85, 140]. This finding contradicts earlier studies linking high desire for control to increased ED risk [40, 111], although some have also reported non-significant associations using similar instruments (EAT-26) [120]. Although significant, the effect was small.

H3.2. Dependency avoidance (*M* = 4.29, SD = 1.49) was not a significant predictor of ED-related behaviours (*β* = 0.33, *p* = 0.226) after controlling for sex and age. However, both sex (*β* = 3.68***, *p* < 0.001) and age (*β* = 0.75**, *p* = 0.002) remained significantly associated with ED symptomatology, as has been demonstrated in other studies [79, 97, 119]. Nevertheless, some research suggests that this avoidant pattern may not be directly involved in general ED symptomatology but may instead be linked to specific clinical subtypes, such as restrictive anorexia nervosa or ED profiles with anxious/obsessive comorbidity [101].

H3.3. Control over preparation/prevention of tasks (*M* = 5.05, SD = 1.51) also showed no statistically significant effect on ED-related behaviours (*β* = −0.005, SE = 0.491, *p* = 0.354). Again, sex (*p* < 0.001) and age (*p* = 0.002) remained significant, with higher scores in females and modest increases with age, aligning with established demographic patterns in ED research [3, 85]. This finding supports evidence that, although perfectionistic and controlling styles are common in ED patients, their predictive power depends on interactions with other variables [6, 40].

## Discussion

This study explored, from a multidimensional perspective, the correlational associations between perfectionism, cognitive flexibility (CF), desire for control (DC), and ED-related behaviours in a cross-sectional, non-clinical sample. Findings are associative, not causal, and should not be directly extrapolated to clinical populations.

All dimensions of perfectionism, except organisation and parental expectations, were associated with higher ED-related behaviours, in accordance with previous research [7, 32, 103]. Hawkins et al. [52] proposed that this discrepancy may be due to the existence of two groups, i.e., healthy and unhealthy perfectionists, with the former typically scoring higher on the organisation dimension. Likewise, Piotrowski and Bojanowska [103] suggested that this subdimension is less strongly associated with ED risk than others. Nevertheless, it remains in use and is still considered by some authors to be a core component of perfectionism [118].

In the present study, concern over mistakes was the subdimension most strongly associated with increased ED behaviours, followed by personal standards and parental criticism. These dimensions have received more consistent empirical support [8, 103, 126]. Regarding doubts about actions, our findings highlight its association with ED-related behaviours. Individuals who frequently question their decisions tend to exhibit more pronounced eating symptomatology, suggesting that a cognitive style marked by insecurity and constant self-evaluation may maintain dysfunctional eating thoughts and behaviours. These findings align with prior research linking indecisiveness and maladaptive perfectionism to greater ED vulnerability [14, 57], highlighting the need to address these patterns in both prevention and non-clinical intervention strategies.

With respect to CF, we found that the avoidance and certainty subdimensions were related to an increased in ED behaviours, in agreement with findings by Jo et al. [60]. In the case of avoidance, lower scores (i.e., greater avoidance) indicated higher cognitive inflexibility. This has been conceptualised as a trait of psychological inflexibility [63, 82], although some studies argue that they are distinct constructs rather than opposite ends of a single dimension [109]. The certainty subdimension (need for predictability and structure) showed a weaker but positive association with ED symptoms. Such cognitive rigidity can promote inflexible, restrictive, or ritualistic behaviours, commonly observed in EDs. These findings align with Jo et al. [60], who found that psychological inflexibility (expressed as difficulty remaining present-focused) is associated with disordered eating. The certainty subdimension may be understood within this broader framework, as an excessive pursuit of certainty could impair adaptability and increase vulnerability to problematic eating behaviours. These results also find conceptual support in mindfulness-based intervention models [72], although their long-term effectiveness remains debated [89]. While interventions such as Acceptance and Commitment Therapy (ACT) propose that greater psychological acceptance can serve as a protective factor against maladaptive behaviours [37, 110], our data suggest that, at least in non-clinical populations, acceptance alone does not appear to exert a direct effect on ED-related behaviours.

Thirdly, regarding DC, our findings partially diverge from previous studies [65, 111]. Of the three subdimensions evaluated, only general desire for control was significantly and negatively associated with ED symptoms: participants with lower general desire for control showed higher levels of disordered eating. This finding challenges the common assumption that high DC is a risk factor for EDs and instead suggests that lower levels of general control may be associated with greater vulnerability. This contrasts with the traditional view that excessive control is central to EDs, particularly in cases of anorexia nervosa [143]. As Brown et al. [13] pointed out, intolerance of uncertainty plays an important role in maintaining EDs, acting as a mediating variable. In this context, a desire for control that fails to translate into effective regulation may be counterproductive, leading to rigid or compensatory behaviours. However, empirical research on this trait and its relationship with EDs remains limited [83].

Additional literature highlights the complexity of this relationship. Sassaroli et al. [112], using an alternative control measure, found lower perceived control associated with disordered eating behaviours. Tiggemann and Raven [133], analysing DC multidimensionally, found that fear of losing self-control was the strongest predictor, with women with ED reporting lower internal control, which may help contextualize our findings. Froreich et al. [40], employing the Burger and Cooper scale as in the present study, also did not find a relationship between general DC and ED behaviours, and proposed that this may be because none of the items refer directly to eating behaviours, supporting our interpretation. Most notably, Murray et al. [96], in a clinical sample, reported that general measures of control did not consistently relate to ED symptomatology, emphasizing that the association between control and disordered eating may be context and subtype dependent. These observations underscore that perfectionism, CF, and DC interact in complex ways, and that their combined assessment may provide a more accurate model of ED vulnerability than evaluating each trait separately [45, 70, 113]. As well, divergent results may reflect sample-specific dynamics, covariate suppression, or measurement characteristics in this non-clinical population. Although some associations were statistically significant, effect sizes and explained variance were generally modest, indicating that other unmeasured factors likely contribute to eating-related behaviours.

The other two subdimensions (dependency avoidance and control over preparation/prevention of tasks) were not significantly associated with ED behaviours. This suggests that not all forms of control have the same explanatory value in this context. In fact, recent research [47, 98, 120] has emphasised that the role of psychological control in EDs depends on the interaction of multiple factors. While desire for control appears to be strongly related to EDs [64], our results indicate that individuals with lower need for autonomy may be more prone to disordered eating behaviours. This dimensional complexity of DC highlights the need for further conceptual clarification, distinguishing between adaptive and maladaptive forms depending on context and population. This aligns with the view that multidimensional profiles, rather than single traits, are necessary to explain vulnerability to EDs [91, 135].

Additionally, significant effects of sex and age were observed: female sex was associated with higher levels of ED symptomatology, in line with prior studies identifying it as a key risk factor [71, 124], reflecting the combined influence of sociocultural pressures, hormonal fluctuations, and genetic factors on ED vulnerability in females [23, 136]. Similarly, a positive relationship between age and ED symptoms was detected, potentially reflecting developmental or contextual factors. Longitudinal research shows that ED symptom trajectories increase from early adolescence into young adulthood, with developmental transitions such as puberty and psychosocial changes interacting with personality traits to shape vulnerability [90, 119]. Furthermore, personality traits related to obsessive–compulsive tendencies demonstrate moderate stability across adolescence and early adulthood, and their persistence may contribute to stable associations with eating-related behaviours across age groups [45, 91, 135]. This suggests that stable personality dispositions can interact with developmental and contextual factors to influence ED symptomatology over time.

### Strengths and limitations

This study has limitations. The sample included only young adults, limiting generalisability. No objective measures, such as BMI, were collected, introducing possible self-report bias [25]. The cross-sectional design precludes causal inference. All measures were self-reported in a non-clinical sample, which may inflate associations and limit construct validity, reducing direct applicability to clinical populations. Future research should use multi-method, multi-informant approaches and include clinical samples to strengthen validity and generalisability.

Participation was voluntary, potentially introducing selection bias. Some instruments had few items per subdimension, reducing sensitivity to subtle differences. The cross-sectional self-report design does not capture situational or environmental influences, such as acute stress or social context. The EAT-26 may overestimate symptoms in non-clinical samples, so findings primarily reflect eating behaviours rather than diagnostic risk. Regarding the Desire for Control Scale, item removal and factor adjustments enhanced internal consistency but may reduce generalisability, highlighting the need for cross-validation. Approximately 20% of data were missing, and although listwise deletion was used, this may affect representativeness; future studies could apply multiple imputation or full information maximum likelihood.

Future studies should include broader age ranges to examine how these personality traits influence ED behaviours across the lifespan. Questions remain regarding the nature, stability, and developmental trajectories of traits, particularly CF and DC, and how they are best conceptualised. Research design improvements, including greater methodological rigour and consistent validation of assessment tools, are needed. Future work should also consider other established risk factors not included here, such as body dissatisfaction or low self-esteem. While evidence exists for these variables individually, their joint interaction remains underexplored [5, 21, 62, 85]. Interaction effects of sex and age with obsessive–compulsive personality traits were not tested. Future research should examine these interactions to achieve a more developmentally and gender-sensitive understanding of eating-related behaviours. As the sample came from a single region of Spain, replication in diverse populations is needed to improve external validity.

### What is already known about this topic?

Perfectionism and parental factors have been consistently associated with the risk of developing eating disorders. Previous studies have shown that traits such as concern about mistakes or high parental expectations may act as vulnerability factors.

### What does this study add?

In conclusion, our findings highlight the importance of considering obsessive–compulsive personality traits jointly when examining their relation to eating-related behaviours, including perfectionism, DC, and CF in a multidimensional manner. The results support the view that combined profiles of these traits better explain variability in eating-related behaviours than examining traits individually [45, 91, 113, 135]. A nuanced understanding of these profiles can inform non-clinical prevention and intervention approaches, without assuming direct clinical applicability [31, 46, 67, 75].

## Supplementary Information

Below is the link to the electronic supplementary material.

Supplementary file 1.

## Data Availability

Data and materials are available upon request from the authors.
