# Supplementary material for: Relationship between obsessive personality traits and eating disorders
Source: Eat Weight Disord. 2026 Mar 12;31(1):48. doi: 10.1007/s40519-026-01829-5 (PMC13216079; doi:10.1007/s40519-026-01829-5)
Supplement: Supplementary file 1 [file 40519_2026_1829_MOESM1_ESM.docx]

Supplementary material for the article:

Relationship between Obsessive Personality Traits and Eating Disorders

Triguero-López, María J.^1^, Moreno-García, M. Inmaculada^1^ y Morales-Ortiz, Manuel^2^

^1^Department of Personality, Psychological Assessment, and Treatment, Faculty of Psychology, University of Seville, Calle Camilo José Cela, 41018, Seville, Spain

^2^Department of Experimental Psychology, Faculty of Psychology, University of Seville, Calle Camilo José Cela, 41018, Seville, Spain

August 25, 2025

**Table S1**

*Age and sex of participants*

| Age | n | Sex | |
| --- | --- | --- | --- |
|  |  | Male | Female |
| 18 | 227 | 64 | 162 |
| 19 | 209 | 126 | 83 |
| 20 | 178 | 105 | 73 |
| 21 | 91 | 55 | 36 |
| 22 | 45 | 12 | 33 |
| 23 | 68 | 47 | 21 |
| 24 | 36 | 19 | 17 |
| 25 | 36 | 26 | 10 |

**Table S2**

*Descriptive statistics of the data obtained*

| Items | N | Lost Values | Average | Median | Standard deviation | Minimum | Maximum |
| --- | --- | --- | --- | --- | --- | --- | --- |
| Age | 869 | 21 | 20 | 19 | 1.968 | 18 | 25 |
| ED1 | 890 | 0 | 3.49 | 3 | 1.945 | 1 | 7 |
| ED2 | 890 | 0 | 2.27 | 2 | 1.454 | 1 | 7 |
| ED3 | 885 | 5 | 2.72 | 2 | 1.551 | 1 | 7 |
| ED4 | 885 | 5 | 2.80 | 2 | 1.932 | 1 | 7 |
| ED5 | 890 | 0 | 2.09 | 1 | 1.497 | 1 | 7 |
| ED6 | 890 | 0 | 2.17 | 1 | 1.649 | 1 | 7 |
| ED7 | 882 | 8 | 2.79 | 2 | 1.845 | 1 | 7 |
| ED8 | 885 | 5 | 3.18 | 3 | 1.872 | 1 | 7 |
| ED9 | 890 | 0 | 3.15 | 3 | 1.872 | 1 | 7 |
| ED10 | 878 | 12 | 2.84 | 3 | 1.684 | 1 | 7 |
| ED11 | 886 | 4 | 2.02 | 1 | 1.582 | 1 | 7 |
| ED12 | 890 | 0 | 2.33 | 2 | 1.634 | 1 | 7 |
| P1_PEX | 888 | 2 | 3.47 | 4 | 1.770 | 1 | 7 |
| P2_ORG | 888 | 2 | 5.14 | 5 | 1.519 | 1 | 7 |
| P3_PC | 883 | 7 | 2.77 | 2 | 1.857 | 1 | 7 |
| P4_PS | 886 | 4 | 3.60 | 4 | 1.970 | 1 | 7 |
| P5_PC | 881 | 9 | 2.49 | 2 | 1.761 | 1 | 7 |
| P6_PS | 881 | 9 | 4.70 | 5 | 1.726 | 1 | 7 |
| P7_ORG | 880 | 10 | 4.85 | 5 | 1.670 | 1 | 7 |

**Table S2**

*Descriptive statistics of the data obtained (continuation)*

|  | N | Lost Values | | Average | | Median | | Standard deviation | | Minimum | | Maximum | |
| --- | --- | --- | --- | --- | --- | --- | --- | --- | --- | --- | --- | --- | --- |
| P8_ORG | 881 | | 9 | | 5.45 | | 6 | | 1.401 | | 1 | | 7 |
| P9_CM | 887 | | 3 | | 2.93 | | 2 | | 1.859 | | 1 | | 7 |
| P10_PEX | 885 | | 5 | | 3.42 | | 3 | | 2.041 | | 1 | | 7 |
| P11_PS | 879 | | 11 | | 3.94 | | 4 | | 1.887 | | 1 | | 7 |
| P12_CM | 888 | | 2 | | 2.89 | | 2 | | 1.823 | | 1 | | 7 |
| P13_CM | 884 | | 6 | | 2.72 | | 2 | | 1.741 | | 1 | | 7 |
| P14_CM | 881 | | 9 | | 2.93 | | 2 | | 1.869 | | 1 | | 7 |
| P15_PS | 882 | | 8 | | 3.49 | | 4 | | 1.828 | | 1 | | 7 |
| P16_PC | 885 | | 5 | | 2.86 | | 2 | | 1.902 | | 1 | | 7 |
| P17_PS | 885 | | 5 | | 4.65 | | 5 | | 1.892 | | 1 | | 7 |
| P18_PS | 881 | | 9 | | 3.79 | | 4 | | 1.947 | | 1 | | 7 |
| P19_ORG | 884 | | 6 | | 5.48 | | 6 | | 1.435 | | 1 | | 7 |
| P20_DA | 881 | | 9 | | 4.31 | | 4 | | 1.655 | | 1 | | 7 |
| P21_PC | 887 | | 3 | | 2.73 | | 2 | | 1.880 | | 1 | | 7 |
| DESCON1 | 885 | | 5 | | 4.04 | | 4 | | 1.717 | | 1 | | 7 |
| DESCON2 | 881 | | 9 | | 4.79 | | 5 | | 1.840 | | 1 | | 7 |
| DESCON3 | 882 | | 8 | | 5.64 | | 6 | | 1.348 | | 1 | | 7 |
| DESCON4 | 878 | | 12 | | 5.79 | | 6 | | 1.360 | | 1 | | 7 |
| DESCON5 | 890 | | 0 | | 4.60 | | 5 | | 1.495 | | 1 | | 7 |
| DESCON6 | 890 | | 0 | | 4.31 | | 4 | | 1.906 | | 1 | | 7 |

**Table S2**

*Descriptive statistics of the data obtained (continuation)*

|  | N | Lost Values | | Average | | Median | | Standard Deviation | | Minimum | | Maximum | |
| --- | --- | --- | --- | --- | --- | --- | --- | --- | --- | --- | --- | --- | --- |
| DESCON7 | 890 | | 0 | | 5.88 | | 6 | | 1.236 | | 1 | | 7 |
| DESCON8 | 882 | | 8 | | 5.53 | | 6 | | 1.406 | | 1 | | 7 |
| DESCON9 | 852 | | 38 | | 5.22 | | 6 | | 1.891 | | 1 | | 7 |
| DESCON10 | 890 | | 0 | | 4.59 | | 5 | | 1.666 | | 1 | | 7 |
| A1 | 890 | | 0 | | 5.23 | | 6 | | 1.609 | | 1 | | 7 |
| A2 | 890 | | 0 | | 4.92 | | 5 | | 1.741 | | 1 | | 7 |
| CER3 | 890 | | 0 | | 4.54 | | 5 | | 1.647 | | 1 | | 7 |
| CER4 | 890 | | 0 | | 3.01 | | 3 | | 1.813 | | 1 | | 7 |
| CER5 | 890 | | 0 | | 3.84 | | 4 | | 1.882 | | 1 | | 7 |
| CER6 | 890 | | 0 | | 3.38 | | 3 | | 1.821 | | 1 | | 7 |
| AC7 | 890 | | 0 | | 4.89 | | 5 | | 1.538 | | 1 | | 7 |
| AC8 | 890 | | 0 | | 4.65 | | 5 | | 1.629 | | 1 | | 7 |

*Note.* ED (ítems from EAT questionnaire), P (items from perfectionism questionnaire): CM (Concern over Mistakes), PS (Personal Standards), PEX (Parental Expectations), PC (Parental Criticism), DA (Doubts about one’s Actions), ORG (Organisation). Items of flexibility questionnaire: A (Avoidance), CER (Certainty), AC (Acceptance), DESCON (ítems from Desire for Control questionnaire).

**Table S3**

*Spearman correlations*

|  | ED | p |
| --- | --- | --- |
| CM | 0.303^***^ | <.001 |
| PS | 0.228^***^ | <.001 |
| PEX | 0.061 | 0.106 |
| PC | 0.233^***^ | <.001 |
| DA | 0.119^**^ | 0.001 |
| ORG | 0.041 | 0.275 |
| A | -0.205^***^ | <.001 |
| CER | 0.151^***^ | <.001 |
| AC | 0.015 | 0.684 |
| GDC | -0.125^***^ | <.001 |
| AD | 0.058 | 0.125 |
| CPP | 0.125 | 0.237 |

*Note.* ^*^ p < .05; ^**^ p < .01; ^***^ p < .001. ED (ED-related behaviours), CM (Concern over Mistakes), PS (Personal Standards), PEX (Parental Expectations), PC (Parental Criticism), DA (Doubts about one’s Actions), ORG (Organisation), A (Avoidance), CER (Certainty), AC (Acceptance), GDC (General Desire for Control), AD (Avoidance of Dependency), CPP (Control over Preparation/Prevention of Tasks).
